# Retinal nerve fiber layer in frontotemporal lobar degeneration and amyotrophic lateral sclerosis

**DOI:** 10.3389/fnins.2022.964715

**Published:** 2022-10-06

**Authors:** Bryan M. Wong, Christopher Hudson, Emily Snook, Faryan Tayyari, Hyejung Jung, Malcolm A. Binns, Saba Samet, Richard W. Cheng, Carmen Balian, Efrem D. Mandelcorn, Edward Margolin, Elizabeth Finger, Sandra E. Black, David F. Tang-Wai, Lorne Zinman, Brian Tan, Wendy Lou, Mario Masellis, Agessandro Abrahao, Andrew Frank, Derek Beaton, Kelly M. Sunderland, Stephen R. Arnott, ONDRI Investigators, Sabrina Adamo, Maria Carmela Tartaglia, Wendy V. Hatch

**Affiliations:** ^1^Faculty of Medicine, University of Toronto, Toronto, ON, Canada; ^2^Department of Ophthalmology and Vision Sciences, University of Toronto, Toronto, ON, Canada; ^3^School of Optometry and Vision Science, University of Waterloo, Waterloo, ON, Canada; ^4^Kensington Eye Institute, Toronto, ON, Canada; ^5^Dalla Lana School of Public Health, University of Toronto, Toronto, ON, Canada; ^6^Rotman Research Institute, Baycrest Health Sciences, Toronto, ON, Canada; ^7^Department of Clinical Neurological Sciences, Western University, London, ON, Canada; ^8^Department of Medicine (Neurology), Sunnybrook Health Sciences Centre, Hurvitz Brain Sciences Research Program, Sunnybrook Research Institute, University of Toronto, Toronto, ON, Canada; ^9^Department of Medicine, Division of Neurology, University of Toronto, Toronto, ON, Canada; ^10^Krembil Brain Institute, University Health Network Memory Clinic, Toronto, ON, Canada; ^11^Bruyere Research Institute, University of Ottawa, Ottawa, ON, Canada

**Keywords:** retinal nerve fibre layer, optical coherence tomography, tauopathy, TDP-43 proteinopathy, frontotemporal lobar degeneration, amyotrophic lateral sclerosis

## Abstract

**Purpose:**

Tauopathy and transactive response DNA binding protein 43 (TDP-43) proteinopathy are associated with neurodegenerative diseases. These proteinopathies are difficult to detect *in vivo*. This study examined if spectral-domain optical coherence tomography (SD-OCT) can differentiate *in vivo* the difference in peripapillary retinal nerve fibre layer (pRNFL) thickness and macular retinal thickness between participants with presumed tauopathy (progressive supranuclear palsy) and those with presumed TDP-43 proteinopathy (amyotrophic lateral sclerosis and semantic variant primary progressive aphasia).

**Study design:**

Prospective, multi-centre, observational study.

**Materials and methods:**

pRNFL and macular SD-OCT images were acquired in both eyes of each participant using Heidelberg Spectralis SD-OCT. Global and pRNFL thickness in 6 sectors were analyzed, as well as macular thickness in a central 1 mm diameter zone and 4 surrounding sectors. Linear mixed model methods adjusting for baseline differences between groups were used to compare the two groups with respect to pRNFL and macular thickness.

**Results:**

A significant difference was found in mean pRNFL thickness between groups, with the TDP-43 group (*n* = 28 eyes) having a significantly thinner pRNFL in the temporal sector than the tauopathy group (*n* = 9 eyes; mean difference = 15.46 μm, SE = 6.98, *p* = 0.046), which was not significant after adjusting for multiple comparisons. No other significant differences were found between groups for pRNFL or macular thickness.

**Conclusion:**

The finding that the temporal pRNFL in the TDP-43 group was on average 15.46 μm thinner could potentially have clinical significance. Future work with larger sample sizes, longitudinal studies, and at the level of retinal sublayers will help to determine the utility of SD-OCT to differentiate between these two proteinopathies.

## Introduction

Frontotemporal dementia (FTD) encompasses a heterogeneous group of clinical syndromes characterized by frontotemporal lobar degeneration (FTLD) and prominent changes in cognition, behavior and motor function. After Alzheimer’s disease (AD), it is the second-most common form of dementia in people under 65 years of age (Hodges et al., 2003; Knopman and Roberts, 2011). Pathologically, FTLD is associated with two predominant proteinopathies: tauopathy and transactive response DNA-binding protein 43 (TDP-43) proteinopathy (Irwin et al., 2015). Some FTLD-related syndromes, such as progressive supranuclear palsy (PSP), are reliably associated with tauopathy (Höglinger et al., 2017; Kim et al., 2019), while semantic variant primary progressive aphasia (svPPA) is almost always associated with TDP-43 proteinopathy (Irwin et al., 2015). Additional subtypes such as behavioral variant FTD (bvFTD), non-fluent variant PPA (nfvPPA) and corticobasal syndrome (CBS) are associated with both proteinopathies (Olney et al., 2017). FTLD-tau and FTLD-TDP-43 are difficult to detect *in vivo* (Irwin et al., 2015).

Amyotrophic lateral sclerosis (ALS) is a motor neuron degenerative disorder characterized by muscle weakness and paralysis (Rojas et al., 2020). It can also be associated with cognitive and behavioral alterations. ALS is also reliably associated with TDP-43 as its main pathological mechanism (Brettschneider et al., 2013; Neumann, 2013; Masala et al., 2018). ALS and some forms of FTLD have overlapping pathology and so are considered to be on the same disease spectrum (Mackenzie and Feldman, 2005).

Spectral-domain optical coherence tomography (SD-OCT) is a clinical tool routinely used to obtain rapid, non-invasive three-dimensional scans of ocular structures including the retina and optic nerve. Since the retina and optic nerve extend from the diencephalon, they are considered parts of the central nervous system. Accordingly, the potential for SD-OCT to detect biomarkers of neurodegenerative disease has been explored (Kirbas et al., 2013; Doustar et al., 2017; Ferrari et al., 2017; Wong et al., 2019).

Although some studies have found thinning in the peripapillary retinal nerve fiber layer (pRNFL; Stemplewitz et al., 2017; Sevim et al., 2018), macula (Stemplewitz et al., 2017; Sevim et al., 2018), and macular sublayers (Kim et al., 2017, 2019; Sevim et al., 2018) in patients with FTD and its subtypes, there is no current consensus on how specifically these structures are affected. Similarly in patients with ALS, although several studies reported a thinner global (average) pRNFL compared to controls (Ringelstein et al., 2014; Mukherjee et al., 2017; Rohani et al., 2018; Rojas et al., 2020), their results differ with respect to how the macula and pRNFL sectors are affected (Roth et al., 2013; Mukherjee et al., 2017; Rohani et al., 2018; Rojas et al., 2019). Furthermore, there is a paucity of research that compares retinal biomarkers based on proteinopathy rather than clinical phenotypes.

Previous studies have emphasized the need to investigate biomarkers to help differentiate tauopathy from TDP-43 proteinopathy (Irwin et al., 2015; Kim et al., 2017). Since PSP is usually associated with tauopathy, while svPPA and ALS are associated with TDP-43 proteinopathy, in this study, pRNFL and macular thickness were used to compare patients with PSP (presumed tauopathy) to those with svPPA or ALS (presumed TDP-43 proteinopathy). Hereafter, presumed tauopathy and presumed TDP-43 proteinopathy will be referred to as tauopathy and TDP-43 proteinopathy, respectively. We hypothesize differences in pRNFL and macular thickness could support the use of SD-OCT to differentiate between these two proteinopathies.

## Materials and methods

This was an analysis of a prospective, multi-center, observational study known as the Ontario Neurodegenerative Disease Research Initiative (ONDRI; Farhan et al., 2017). Baseline characteristics of the full ONDRI cohort are summarized in a study by Sunderland and colleagues (Sunderland et al., 2022). Participants diagnosed with FTLD-related syndromes or ALS were recruited from the Toronto Western Hospital (Toronto, Canada), Sunnybrook Hospital (Toronto, Canada), Baycrest Hospital (Toronto, Canada), Parkwood Institute (London, Canada), and Élisabeth Bruyère Continuing Care (Ottawa, Canada), between 2014 and 2018. This study was approved by the Research Ethics Boards at the University Health Network, Sunnybrook Research Institute, University of Waterloo, and the University of Western Ontario. The study protocol followed the tenets of the Declaration of Helsinki and participants provided informed consent.

### Inclusion and exclusion criteria

General inclusion and exclusion criteria for the ONDRI cohort were outlined by Farhan and co-workers (Farhan et al., 2017). The study included four neurodegenerative diseases (FTLD-related syndromes (PSP, svPPA, bvFTD, nfvPPA, and CBS), ALS, AD/mild cognitive impairment, Parkinson’s disease) and cerebrovascular disease (Farhan et al., 2017). Exclusion criteria specific to the SD-OCT retinal imaging platform within ONDRI included poorly controlled diabetes mellitus, glaucoma or glaucoma suspect (defined as intraocular pressure (IOP) greater than 22 mmHg in either eye, IOP difference between eyes > 5 mmHg, optic nerve head cup-to-disk ratio (C/D) ≥ 0.7, or C/D difference between eyes > 0.2), and other optic neuropathies or maculopathies (such as exudative age related macular degeneration) identified on inspection of fundus photographs by an ophthalmologist (EM, EDM) masked to the participant’s underlying diagnosis. SD-OCT images with a quality score of less than 20 were excluded. Upon inspection by expert observers (WVH, FT, CH, CB, RC) and consultation with ophthalmologists when necessary, SD-OCT images were excluded if macular thickness or pRNFL thickness could be affected by pathologies or structural anomalies including tilted optic disks. Participants with a history of stroke were also excluded from the analysis as potential retrograde degeneration in the retina could affect thickness measurements.

### Procedure

Peripapillary RNFL and macular SD-OCT scans were acquired in both eyes of each participant using the Heidelberg Spectralis SD-OCT, acquisition software version 6.0.13.0 (Heidelberg Engineering GmbH, Heidelberg, Germany), and analyzed with the Heidelberg Eye Explorer software (HEYEX version 6.3.4.0). Prior to analyses, each scan was inspected for any software-generated retinal segmentation lines that did not accurately delineate the internal limiting membrane and Bruch’s membrane, and were manually corrected by a trained observer.

**FIGURE 1:**
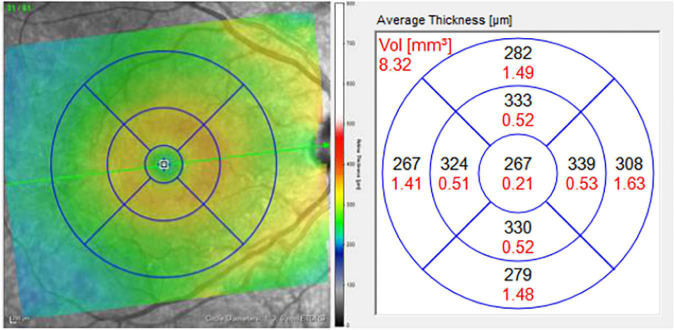
Macular grid with 1, 3, and 6 mm diameter circles centered on the foveola **(left)**. Measurements for average thickness (black) in areas of each sector of the macula **(right)**. Only the central five macular sectors were analyzed in this study: central macula, and the inner superior, inferior, nasal, and temporal sectors, bordered by the 3 mm diameter circle.

**FIGURE 2:**
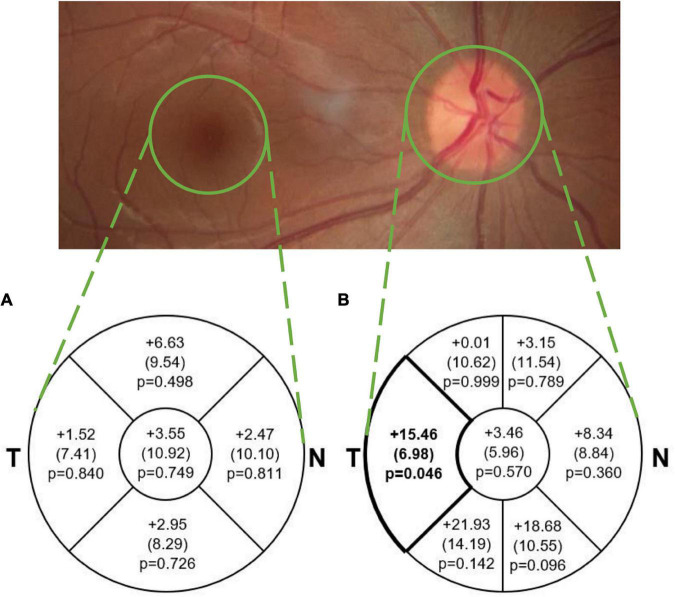
Mean (SE) difference (tauopathy – TDP43, μm) in average **(A)** macular sectoral thickness and **(B)** pRNFL sectoral and global thickness. The difference in pRNFL thickness between groups in the temporal sector (*p* < 0.05; medium effect size of 0.61) is illustrated in bold. This difference was not significant after adjusting for multiple comparisons. Note that average thickness in the macular scan is *within the area* of each sector and represents total retinal thickness, while in the pRNFL scan it is *along the circular line* of each sector and represents RNFL thickness only. T, Temporal; N, Nasal.

For each pRNFL OCT scan, seven pRNFL thickness measurements were captured: global (average) and 6 sectors (temporal, superior-temporal, inferior-temporal, nasal, superior-nasal, and inferior-nasal) using a B-scan with a diameter of 3.5 mm centered on the middle of the optic nerve head (Figure 1). For each macular (posterior pole) OCT image, a grid with concentric circles of 1 and 3 mm diameters was centered on the fovea (Figure 2). Average retinal thickness was measured in the central 1 mm diameter zone, as well as 4 surrounding sectors (superior, inferior, temporal, and nasal) within the 3 mm diameter circular grid.

### Analysis

PSP is usually associated with tauopathy, while svPPA and ALS are associated with TDP-43 (Irwin et al., 2015; Höglinger et al., 2017; Kim et al., 2019). Any participants with other clinical subtypes of FTD that involve both tauopathy and TDP-43 proteinopathies (i.e., CBS, bvFTD, and nfPPA) were excluded.

A linear mixed model adjusting for fixed effects of eye, age, and sex, and accounting for within-subject correlation, was used to compare both pRNFL thickness and macular retinal thickness between eyes in subjects with tauopathy to those with TDP-43 proteinopathy. The unstructured covariance matrix was used for errors. A Bonferroni adjustment was employed to control for multiple comparisons which yielded a threshold of significance of 0.05/12 = 0.0042, and controls the familywise error rate at the level of 0.05. SAS statistical software (version 9.4, SAS Institute, Cary, NC) was used for analysis.

A sensitivity analysis with a linear mixed repeated measures model adjusting for age, sex, and eye was also performed, excluding the 3 svPPA participants in the TDP43 group, leaving only those with ALS in the group.

An outlier analysis was also performed on the svPPA group to assess whether it could influence the results of the TDP43 group.

Furthermore, in the 11 subjects with ALS, a Wilcoxon signed rank test was performed to compare the left and right eyes.

## Results

The final dataset included 9 eyes of 5 subjects (3 female) in the tauopathy group (with a clinical diagnosis of PSP) and 28 eyes of 14 subjects (4 female) in the TDP-43 group (3 with a clinical diagnosis of svPPA and 11 with ALS). Baseline characteristics are described in Table 1. The groups were not matched for age or sex. There were 9 eyes in the tauopathy group; there were 8 eyes with pRNFL scans, and a different 8 eyes with macular scans.

**TABLE 1:** Participant demographics.

	Tauopathy (*n* = 9 eyes, 5 subjects)	TDP-43 Proteinopathy (*n* = 28 eyes, 14 subjects)
**Clinical diagnosis, *n*** PSP svPPA ALS	5 N/A N/A	N/A 3 11
Mean age, years (SD)	72.7 (5.2)	62.1 (9.8)
**Sex, *n* (%)** Male Female	2 (40%) 3 (60%)	10 (71.4%) 4 (28.6%)
Eyes with pRNFL scans	8	27
Eyes with macular scans	8	28

PSP, progressive supranuclear palsy; svPPA, semantic variant primary progressive aphasia; ALS, amyotrophic lateral sclerosis.

For the retinal thickness (RT) scan in subjects with FTLD, 23 eyes in 12 subjects were excluded due to fixation difficulties during their scan, and 9 eyes in 5 subjects were excluded due to pathologies including AMD, macular hole, lamellar hole, glaucoma, and uncontrolled diabetes (HbA1c > 7.5).

For the pRNFL scan in subjects with FTLD, 15 eyes in 8 subjects were excluded due to fixation difficulties during their scan, and 6 eyes in 3 subjects were excluded due to pathologies including AMD, lamellar hole, cupped optic disk, and uncontrolled diabetes (HbA1c > 7.5).

Of the 30 subjects who met criteria In the FTLD group, only 8 subjects were used (5 in the PSP and 3 in the svPPA group), as the other FTLD subjects did not meet the criteria for a diagnosis of PSP or svPPA.

For the RT and pRNFL scans in subjects with ALS, in a *post hoc* analysis, 2 subjects (4 eyes) with tilted nerves and 1 subject (2 eyes) with a history of stroke were excluded since they could potentially exhibit abnormal pRNFL thicknesses.

Mean pRNFL and macular thickness values in each group are described in Table 2, and mean differences between groups are illustrated in Figure 2. The TDP-43 group demonstrated a thinner pRNFL in the temporal sector than the tauopathy group (mean difference = 15.46 μm, SE = 6.98 (95% CI: 0.32, 30.60), *p* = 0.046) with a medium effect size of 0.61. Based on adjustment for multiple comparisons, this difference is not statistically significant. The differences in global pRNFL thickness between groups and for the other sectors were not significantly different (Figure 2).

**TABLE 2:** Mean peripapillary retinal nerve fibre layer and macular thickness values in tauopathy and TDP-43 proteinopathy groups, adjusted for age and sex.

	Adjusted model*				Non-adjusted model
	Tauopathy (*n* = 9 eyes, 5 subjects)	TDP-43 proteinopathy (*n* = 28 eyes, 14 subjects)	Difference		
	Adjusted mean (95% CI)	Adjusted mean (95% CI)	Coefficient for group (95% CI)	*P*-value**	*P*-value
**pRNFL thickness [μm]**					
Global	104.16 (93.53,114.80)	100.71 (94.77,106.64)	3.46 (–9.23, 16.15)	0.5703	0.6438
Temporal	85.77 (72.98, 98.55)	70.31 (62.42, 78.20)	15.46 (0.32, 30.60)	0.0461	0.0064
Superior-temporal	137.83 (119.12,156.55)	137.83 (127.51,148.14)	0.01 (–22.52, 22.54)	0.9994	0.7989
Inferior-temporal	162.88 (137.70,188.06)	140.95 (125.76,156.15)	21.93 (–8.25, 52.10)	0.1426	0.7864
Nasal	85.97 (70.33,101.61)	77.63 (69.00, 86.25)	8.34 (–10.43, 27.11)	0.3598	0.6358
Superior-nasal	108.27 (86.93,129.60)	105.12 (91.67,118.56)	3.15 (–21.36, 27.66)	0.7885	0.7078
Inferior-nasal	132.13 (113.54,150.72)	113.45 (102.88,124.02)	18.68 (–3.68, 41.04)	0.0956	0.5725
**Macular thickness [μm]**					
Central	275.04 (255.85,294.23)	271.49 (260.73,282.25)	3.55 (–19.61, 26.71)	0.7493	0.7070
Superior	344.08 (327.37,360.79)	337.45 (328.10,346.80)	6.63 (–13.66, 26.91)	0.4976	0.8315
Inferior	337.93 (323.16,352.70)	334.98 (326.54,343.42)	2.95 (–14.62, 20.52)	0.7264	0.9516
Temporal	326.66 (313.46,339.85)	325.13 (317.44,332.83)	1.52 (–14.10, 17.15)	0.8397	0.6632
Nasal	344.06 (325.98,362.13)	341.59 (331.38,351.79)	2.47 (–19.33, 24.26)	0.8106	0.7918

*Model was adjusted for age, sex, and eye.

**Significance level after adjusting for multiple comparisons (Bonferroni correction) is 0.004 (=0.05/12). None of the differences were significant at the 0.4% level.

No statistically significant differences were found between the groups for mean retinal thickness in the central 1 mm diameter macular zone between the tauopathy (275.04 μm, SE = 9.06) and TDP-43 (271.49 μm, SE = 5.05) groups (*p* = 0.749). Similarly, no significant differences were found in the surrounding 4 sectors within the 3 mm diameter grid of the macula between the groups.

The *post hoc* sensitivity analysis that excluded the svPPA participants, allowing for a comparison of the tauopathy group to the ALS group, did not yield any new findings that were significant after controlling for multiple comparisons. Additionally, the outlier analysis found no outliers in the 3 svPPA participants in terms of age, macular, or pRNFL thickness. Comparison of the left and right eyes in those with ALS showed that the temporal pRNFL was thinner in the left eye compared to the right eye.

## Discussion

This study found that the pRNFL in the temporal sector in the presumed TDP-43 proteinopathy group was on average approximately 15 μm thinner than the presumed tauopathy group (*p* = 0.046). However, this difference was not statistically significant after applying a conservative adjustment for multiple comparisons.

To our knowledge, no studies have compared pRNFL or macular thickness between FTLD-tau and FTLD-TDP-43 proteinopathies. PSP is usually associated with tauopathy, while svPPA and ALS are associated with TDP-43 proteinopathy (Irwin et al., 2015; Höglinger et al., 2017; Kim et al., 2019). Based on this, we compared a group with tauopathy to a group with TDP-43 proteinopathy. Since the pRNFL and retina thins with age even in healthy normal eyes, we adjusted for age in our analysis (Eriksson and Alm, 2009; Celebi and Mirza, 2013). A summary of the findings of previous related studies is shown in Supplementary Table 1. One study found thinning in the temporal pRNFL in ALS subjects compared to healthy controls (Mukherjee et al., 2017). This temporal sector is the same area that was thinner in our TDP-43 group compared to the tau group. Compared to healthy controls, pRNFL thinning has also been reported in subjects with PSP (Stemplewitz et al., 2017), TDP-43 (Ward et al., 2014), a pathologically heterogeneous group of subjects with FTD (Ferrari et al., 2017), and ALS (Ringelstein et al., 2014; Mukherjee et al., 2017; Rohani et al., 2018). In contrast, two groups reported no significant differences in pRNFL thickness between ALS subjects and controls (Roth et al., 2013; Rojas et al., 2019). Our finding in the ALS group of the left pRNFL being thinner than the right is consistent with two studies that found either pRNFL or ganglion cell complex (GCC) thinning in the left eye compared to the right in ALS subjects, which may suggest asymmetric CNS involvement in the disease (Rohani et al., 2018; Rojas et al., 2019).

With regards to macular thickness, this study found no differences between the groups. Kim and co-workers (Kim et al., 2017, 2019) reported no differences in total retinal thickness in the central 3 mm diameter macular zone between participants with FTD and healthy controls. However, they did find the outer retina sublayer to be significantly thinner in the FTD group overall and in the FTLD-Tau subtype (with PSP, CBS or nfPPA) compared to controls (Kim et al., 2017). With longitudinal analysis, they found this outer retina thinning to persist and correlate with disease progression in participants with probable tauopathy (Kim et al., 2019). In this study, only the central macular sectors were analyzed because the density of the retinal ganglion cells (RGCs) is the highest in this area. Degeneration of neurons in these central sectors would be more pronounced compared to sectors that are relatively more peripheral (Wässle et al., 1989).

Three groups reported significant macular changes in proteinopathies compared to healthy controls (Ward et al., 2014; Stemplewitz et al., 2017; Rojas et al., 2019), including reduced macular volume in subjects with progranulin (GRN) mutations (which are associated with TDP-43 neuropathology), and thinning in six macular sectors in subjects with PSP (Stemplewitz et al., 2017). Rojas’ group (Rojas et al., 2019) reported greater macular thicknesses in the inferior and temporal 3 mm sectors in subjects with ALS compared to controls. However, 6 months later, the inferior 3 and 6 mm sectors were both significantly thinner than at baseline. Because the participants in Rojas’ study were at a similar early stage of disease (within 18 months of motor symptom onset) and were then followed over time, this captured a potential evolution of macular thickening followed by thinning, which may illustrate the disease course of ALS in the macula. Furthermore, since previous studies have reported changes to macular sublayers (including the ganglion cell layer, inner nuclear layer, and outer plexiform layer) in these proteinopathies (Ringelstein et al., 2014; Ward et al., 2014; Sevim et al., 2018), differences in sublayer thicknesses between these groups also warrants further investigation.

This study has several strengths. This is the first study to use SD-OCT to compare participants with tauopathy and TDP-43 proteinopathy by using clinical phenotypes (Kim et al., 2017, 2019). The ability to find non-invasive biomarkers to differentiate between tauopathy and TDP-43 pathology is important as proteinopathy-targeted therapies are being developed (Liscic et al., 2020; Yang et al., 2020). A second strength of this study was the rigorous inspection of SD-OCT images and fundus photographs to exclude pathologies unrelated to neurodegeneration that could affect retinal or pRNFL thickness. Large delineation errors on SD-OCT images were corrected to prevent spurious thickness measurements. Images were also inspected for non-pathological structural anomalies that could potentially affect pRNFL thickness measurements, such as tilted optic disks (Brito et al., 2015), and subjects with these anomalies were excluded. Additionally, subjects with a history of stroke were excluded from the analysis as potential retrograde degeneration in the retina could affect thickness measurements. This rigorous inspection and data curation provided a dataset to reliably identify changes in thickness due to these neurodegenerative diseases.

There are limitations to consider in this study. First, the sample size was small, which may make the findings less generalizable. Despite this, we found a non-significant, but medium effect size difference in the temporal pRNFL thickness between groups. This difference of 15.46 μm may be considered clinically significant. Based on this observed difference, a future study with larger sample size will be able to provide sufficient evidence of statistical significance. For example, a future balanced-design study could recruit 152 patients in total that will likely provide a desirable 80% power to detect significant difference in temporal pRNFL thickness at 5% level. Second, there is a need to consider that the various sectors of pRNFL contain retinal arterioles and venules that typically enter and exit the optic nerve head near the 6 and 12 o’clock positions. At this point on the nerve head, the vessel lumen diameters typically range from 80 to 140 μm for arterioles and 100–150 μm for venules. These vessels can pulse and change in diameter and position with the cardiac cycle. Another limitation is that the disease staging for ALS and PSP are classified using different scales. As such, it is difficult to compare the severity of participants with these different diseases from a clinical standpoint. Consequently, variations in retinal vessel diameter and pulsation will add noise to any structural measurement of pRNFL thickness. Furthermore, the clinical subgroup diagnoses of FTLD and ALS were used to define the pathological groups of tauopathy and TDP-43 proteinopathy. Although the clinical subgroups included were reliably associated with either tau or TDP-43, and any subgroups that were known to involve both pathological mechanisms were eliminated to reduce confounding factors, future studies would hopefully include only biomarker proven proteinopathies. Lastly, if ALS progression consists of a change from pRNFL thickening to thinning with an intermediate stage of normal thickness, as Rojas and co-workers suggested, measurements may be affected by including subjects with different disease severities (Rojas et al., 2019). Future work should involve assessing this cohort over time.

In conclusion, this was the first study to compare pRNFL and macular thickness between participants with PSP (tauopathy) and svPPA and ALS (TDP-43 proteinopathy). The thinning in the temporal pRNFL of the TDP-43 group compared to the tau group warrants further investigation to determine whether retinal imaging can help identify and differentiate proteinopathies in neurodegenerative disease. Future work should assess changes in thickness over time and retinal sub-layer thicknesses to assess whether they are also factors that can help differentiate these two pathological mechanisms.

## Data availability statement

The raw data supporting the conclusions of this article will be made available by the authors through request from the Ontario Brain Institute (see ondri.ca).

## Ethics statement

The studies involving human participants were reviewed and approved by the Research Ethics Boards at the University Health Network, Sunnybrook Research Institute, University of Waterloo, and the University of Western Ontario. The patients/participants provided their written informed consent to participate in this study.

## The ONDRI investigators

Sabrina Adamo, Stephen Arnott, Rob Bartha, Derek Beaton, Courtney Berezuk, Alanna Black, Alisia Bonnick, David Breen, Don Brien, Susan Bronskill, Dennis Bulman, Leanne Casaubon, Ying Chen, Marvin Chum, Brian Coe, Ben Cornish, Jane Lawrence Dewar, Roger A. Dixon, Sherif El-Defrawy, Sali M.K. Farhan, Frederico Faria, Julia Fraser, Mahdi Ghani, Barry Greenberg, Hassan Haddad, Wendy Hatch, Melissa Holmes, Chris Hudson, Peter Kleinstiver, Donna Kwan, Elena Leontieva, Brian Levine, Wendy Lou, Efrem D. Mandelcorn, Ed Margolin, Connie Marras, Bill McIlroy, Paula McLaughlin, Manuel Montero Odasso, Doug Munoz, David Munoz, Nuwan Nanayakkara, JB Orange, Miracle Ozzoude, Alicia Peltsch, Pradeep Raamana, Joel Ramirez, Natalie Rashkovan, Angela Roberts, Yanina Sarquis Adamson, Christopher Scott, Michael Strong, Stephen Strothers, Sujeevini Sujanthan, Kelly M. Sunderland, Sean Symons, Faryan Tayyari, Athena Theyers, Angela Troyer, Abiramy Uthirakumaran, Karen Van Ooteghem, John Woulfe, Mojdeh Zamyadi, and Guangyong (GY) Zou.

## Author contributions

BW, CH, ES, FT, HJ, MB, SS, RC, MT, and WH contributed to conception and design of the study. EF, SB, DT-W, LZ, MM, AA, AF, and MT contributed to participant recruitment. BW, CH, FT, CB, MB, RC, EDM, EM, BT, DB, KS, SA, and WH contributed to curation, processing, and/or quality control of the data. BW, CH, HJ, MB, WL, and WH contributed to the statistical analysis. BW wrote the first draft of the manuscript. BW, CH, HJ, MT, and WH wrote sections of the manuscript. All authors contributed to manuscript revision, read, and approved the submitted version.
