# Supplementary material for: Retinal nerve fiber layer in frontotemporal lobar degeneration and amyotrophic lateral sclerosis
Source: Front Neurosci. 2022 Oct 6;16:964715. doi: 10.3389/fnins.2022.964715 (PMC9583385; doi:10.3389/fnins.2022.964715)
Supplement: Supplementary file 1 [file Table_1.DOCX]

**Supplementary Table 1.** Summary of findings from previous studies that evaluated pRNFL or macular thickness in FTD or ALS using OCT. Findings for pRNFL, total macular, and macular sublayer thickness described are compared to healthy controls, unless specified otherwise.
GCL: ganglion cell layer, IPL: inner plexiform layer, INL: inner nuclear layer, OPL: outer plexiform layer, ONL: outer nuclear layer, EZ: ellipsoid zone, IRL: inner retinal layer, ORL: outer retinal layer.

| **Study and year of publication** | **Disease group** | **pRNFL thickness** | **Total macular thickness** | **Macular sublayer thickness** | **Longitudinal findings** |
| --- | --- | --- | --- | --- | --- |
| Ward et al., 2014 | FTD (TDP-43) | ↓ | ↓ volume | ↓ GCL and INL thickness |  |
| Stemplewitz et al., 2017 | PSP | ↓ inferior-nasal and inferior-temporal sectors | ↓ volume  ↓ thickness in 6 sectors |  |  |
| Ferrari et al., 2017 | FTD (bvFTD, nfPPA, svPPA) and Alzheimer’s disease | ↓ globally in FTD compared to mild AD and healthy controls |  | ↓ GCL-IPL in FTD compared to mild AD and healthy controls |  |
| Kim et al., 2017 | FTD (tauopathy) |  | No significant difference | ↓ ORL, ONL, and EZ in FTD compared to healthy controls  ↓ ONL and EZ in tauopathy subgroup compared to healthy controls |  |
| Sevim et al., 2018 | PSP and Parkinson disease (PD) | ↓ superior quadrant |  | ↓ GCL, IPL, INL, IRL compared to PD and healthy controls  ↓ inner and outer macular volume compared to PD and healthy controls | ↓ pRNFL, and macular ONL, IRL, ORL thickness  ↓ foveal, macular, and inner macular volume |
| Kim et al., 2019 | FTD (tauopathy and TDP-43) |  | No significant difference | ↓ ORL, ONL, and EZ in FTD compared to healthy controls  ↓ EZ and ORL in tauopathy subgroup compared to healthy controls  No significant difference in TDP-43 subgroup compared to healthy controls | No significant rate of change of retinal layers in FTD group after 1-2 years  ↓ macular ORL, ONL, and INL thickness in tauopathy subgroup compared to healthy controls after 1-2 years |
|  |  |  |  |  |  |
| Roth et al., 2013 | ALS | No significant difference | No significant difference | No significant difference |  |
| Ringelstein et al., 2014 | ALS | ↓ | ↓ | ↓ INL  ↑ OPL |  |
| Mukherjee et al., 2017 | ALS | ↓ globally, temporal, superior-nasal (right eye), and superior-temporal (left eye) |  |  |  |
| Rohani et al., 2018 | ALS | ↓ globally  ↓ superior and nasal quadrant (left eyes) in ALS compared to healthy controls |  |  |  |
| Rojas et al., 2019 | ALS | No significant difference | ↑ inferior and temporal 3 mm sectors |  | ↓ pRNFL superior and inferior quadrant thickness in ALS group compared to ALS baseline after 6 months  ↓ macular inferior sectors compared to ALS baseline after 6 months |
