# Supplementary material for: Retinal nerve fiber layer in frontotemporal lobar degeneration and amyotrophic lateral sclerosis
Source: Front Neurosci. 2022 Oct 6;16:964715. doi: 10.3389/fnins.2022.964715 (PMC9583385; doi:10.3389/fnins.2022.964715)
Supplement: Supplementary file 2 [file Table_2.DOCX]

**Supplementary Table 2. Optical coherence tomography acquisition and analysis details**

| Study protocol | # of OCT operating sites: 5  # of graders: 5  Timing of OCT compared to other measurements: Delayed  Inclusion criteria: see methods  Exclusion criteria: see methods |
| --- | --- |
| Acquisition device | Manufacturer: Heidelberg  Model: Spectralis  Acquisition software version: 6.0.13.0  Device type: Spectral domain |
| Acquisition settings | Pupils were dilated before examination  Number of devices: 5 |
| Scanning protocol | Location and type of scans: Macular cube and pRNFL circular scans  Scan parameters: Macula (61 horizontal B scans in posterior pole, in a circle with 6 mm radius around the fovea); pRNFL (1 circular scan with diameter of 3.5mm centred on the optic nerve head); eye tracking was used for both scans |
| Funduscopic imaging | Fundus photographs were also used in addition to OCT (regular coloured digital photographs) |
| Postacquisition data selection | Quality control: Only OCT scans with a quality score of 20 or higher were included. SD-OCT images with a quality score of less than 20 were excluded. SD-OCT images were excluded if macular thickness or pRNFL thickness could be affected by pathologies or structural anomalies including tilted optic discs  Postacquisition discard:  FTD RT: *9 eyes in 5 subjects were excluded due to pathologies including AMD, macular hole, lamellar hole, glaucoma, and HbA1c greater than 7.5.*  FTD pRNFL: *6 eyes in 3 subjects were excluded due to pathologies including AMD, lamellar hole, cupped optic disc, and HbA1c greater than 7.5.*  ALS RT: *4 eyes in 2 subjects were excluded due to glaucoma*  ALS pRNFL: *4 eyes in 2 subjects were excluded due to glaucoma* |
| Postacquisition analysis | Processing software: Heidelberg Eye Explorer software (HEYEX version 6.3.4.0)  Retinal layers segmented: Inner limiting membrane (ILM), RNFL, and Bruch’s membrane (BM)  Method of segmentation: automated  Grid used for data extraction: Macula (Circular grid with concentric circles of 1 and 3 mm diameters, centred on the fovea. Average retinal thickness was measured in the central 1 mm diameter zone, as well as 4 surrounding sectors (superior, inferior, temporal, and nasal) within the 3 mm diameter circular grid.  pRNFL: seven pRNFL thickness measurements were captured: global (average) and 6 sectors (temporal, superior-temporal, inferior-temporal, nasal, superior-nasal, and inferior-nasal) using a B-scan with a diameter of 3.5 mm centered on the middle of the optic nerve head |
| Nomenclature/abbreviations | Anatomical structures analyzed:  Macula scan: Total retinal thickness pRNFL scan: pRNFL thickness  Units: Thickness (µm) |
| Statistical approach | Please see Analysis subsection of Methods |
